# piR-61298 promotes colorectal cancer progression through destabilizing p53 by interacting with USP10

**DOI:** 10.7555/JBR.39.20250137

**Published:** 2026-05-21

**Authors:** Shenya Xu, Zhutao Ding, Shuai Ben, Chen Li, Silu Chen, Lingyan Zhao, Shuwei Li, Dongying Gu

**Affiliations:** 1 Department of Oncology, Nanjing First Hospital, Nanjing Medical University, Nanjing, Jiangsu 210006, China; 2 Department of Genetic Toxicology, the Key Laboratory of Modern Toxicology of Ministry of Education, Key Laboratory of Public Health Safety and Emergency Prevention and Control Technology of Higher Education Institutions in Jiangsu Province, Center for Global Health, School of Public Health, Nanjing Medical University, Nanjing, Jiangsu 211166, China; 3 Key Laboratory of Environmental Medicine Engineering, Ministry of Education, School of Public Health, Southeast University, Nanjing, Jiangsu 211189, China

**Keywords:** piRNA, colorectal cancer, USP10, p53, deubiquitination

## Abstract

PIWI-interacting RNAs (piRNAs) are a class of noncoding RNAs primarily found in germ cells. While piRNAs are known to be involved in various cancers, their specific roles in colorectal cancer (CRC) remain unclear. To elucidate the role of piRNAs in CRC, we first analyzed their expression characteristics by sequencing 10 pairs of tumor and adjacent normal tissues. Subsequently, differentially expressed piRNAs were identified through a two-stage reverse transcription-quantitative PCR (RT-qPCR) validation using 20 and 114 pairs of samples. Subcellular localization was assessed through nucleoplasmic separation and immunofluorescence staining assays. RNA pull-down mass spectrometry was employed to identify piRNA-interacting proteins. We identified piR-61298 as a piRNA significantly upregulated in CRC. Functional assays showed that piR-61298 promoted cell proliferation and migration, inhibited apoptosis, and promoted tumor growth. Mechanistically, piR-61298 bound to ubiquitin-specific peptidase 10 (USP10) in the cytoplasm, impairing its deubiquitinating activity toward p53, thereby leading to p53 ubiquitination and degradation. These findings suggest that piR-61298 plays a critical role in CRC progression by disrupting the USP10-p53 axis. Collectively, the current study highlights piR-61298 as a potential therapeutic target, offering a novel approach for CRC treatment by targeting piRNA-mediated regulation.

## Introduction

Colorectal cancer (CRC) is one of the most prevalent malignancies of the digestive system. In 2020, nearly one million new cases of CRC were diagnosed worldwide, resulting in approximately 900000 deaths^[1]^. In China, both the incidence and mortality rates of CRC rank among the top three and remain consistently high^[2]^. Because of its multifactorial nature, CRC is influenced by a variety of mechanisms, including genetic alterations^[3]^, epigenetic modifications^[4]^, and other cumulative factors^[5]^. As a result, the identification of novel biomarkers and therapeutic targets for accurate diagnosis and effective treatment of CRC has become a critical focus of ongoing research.

PIWI-interacting RNAs (piRNAs) are small noncoding RNAs, typically ranging from 24 to 31 nucleotides in length and lacking a well-defined secondary structure^[6]^. The PIWI protein family maintains a highly conserved structure and function across different species^[7]^. PIWI proteins associate with piRNAs to form complexes that play critical roles in silencing transposons, regulating gene expression, and modulating epigenetic and protein functions^[8]^. Recent studies have highlighted the importance of piRNAs in cancer progression. For example, the PIWIL1 protein promotes the metastatic potential of cancer cells in pancreatic ductal adenocarcinoma by activating the anaphase-promoting complex/cyclosome (APC/C) E3 complex^[9]^. Additionally, piR-30473 contributes to the progression of diffuse large B-cell lymphoma and is associated with poor prognosis through N6-methyladenosine RNA methylation^[10]^. However, the role of piRNAs in the progression of CRC remains unclear.

Ubiquitination is a common post-translational modification that is mediated by the coordinated actions of ubiquitin-activating enzymes (E1), ubiquitin-conjugating enzymes (E2), and ubiquitin ligases (E3)^[11]^. This process is crucial for maintaining cellular homeostasis^[12]^. Among these enzymes, E3 ligases play a crucial role in recruiting substrates and catalyzing the transfer of ubiquitin to them^[13]^. Ubiquitin-specific peptidase 10 (USP10), a highly conserved deubiquitinating enzyme, has been implicated in various cancer types^[14]^. However, its role in tumorigenesis depends on its interaction with specific substrates^[15]^. Thus, identifying the substrates of USP10 and understanding the underlying mechanisms are critical for elucidating its role in cancer.

In the present study, we aimed to investigate the role of piRNAs in CRC progression. To this end, we conducted a comprehensive screening and functional analysis of piRNAs dysregulated in CRC tissues. We further explored their interactions with protein partners and examined their impact on key tumor suppressor pathways using molecular and cellular assays.

## Materials and methods

### Study participants

CRC and adjacent tissues were collected from patients with CRC who underwent surgical resection at the First Affiliated Hospital of Nanjing Medical University. The study was reviewed and approved by the Institutional Review Board of Nanjing Medical University and conducted in accordance with the ethical standards set by the institution and national research committees, as well as the principles outlined in the Declaration of Helsinki. All participants provided written informed consent after being fully informed about the study objectives and procedures. Baseline demographic and clinical data for the study population are presented in ***Supplementary Tables 1*** and ***2***. Total RNA was extracted from tumor tissue samples and subjected to transcriptome profiling using next-generation sequencing (NGS) technology to obtain a comprehensive overview of gene expression.

### Cell culture

Human CRC cell lines HCT116 (Cat. #TCH-C185, Hycyte, Suzhou, China) and LoVo (Cat. #TCH-C239, Hycyte) were cultured at 37 ℃ with 5% CO_2_. HCT116 cells were maintained in McCoy's 5A medium (Cat. #16600082, Gibco, Carlsbad, USA), while LoVo cells were cultured in RPMI-1640 medium (Cat. #11875119, Gibco); both media were supplemented with 10% and 20% fetal bovine serum (FBS; Cat. #A5256701, Gibco), respectively. Cells were routinely passaged at 80%–90% confluence using trypsin-EDTA (Cat. #BC-CE-005, Sbjbio, Nanjing, China) and centrifuged at 1000 *g* for three minutes. For cryopreservation, cells were resuspended in freezing medium containing 90% FBS and 10% DMSO and stored at −80 ℃ or in liquid nitrogen.

### RNA isolation and reverse transcription-quantitative PCR (RT-qPCR)

Total RNA was isolated from cultured cells using TRIzol Reagent (Cat. #15596018CN, Invitrogen, Carlsbad, CA, USA) and quantified using the Nanodrop 2000 spectrophotometer (Thermo Fisher Scientific, Waltham, MA, USA). The relative expression levels of piR-61298, *USP10*, and internal control genes were detected using the LightCycler 480 PCR system (Roche, Basel, Switzerland). The geometric mean of *GAPDH* expression served as the reference for standardizing *USP10* expression, while the geometric mean of *U6* expression served as the reference for standardizing piR-61298 expression. The primer sequences are listed in ***Supplementary Table 3***.

### Plasmids, siRNAs, and cell transfection

Overexpression plasmids for piR-61298 and *USP10*, along with empty vectors (negative control, NC) and siRNAs, were designed and synthesized by RiboBio (Guangzhou, China), with the corresponding sequences listed in ***Supplementary Tables 4*** and ***5***. The lentiviral vector carrying piR-61298 was produced by GeneChem (Shanghai, China). Lipofectamine 3000 reagent (Invitrogen) was used for cell transfection. Briefly, cells were seeded in culture dishes 24 h before transfection to achieve 60%–80% confluence at the time of transfection. The lipid-DNA complex was prepared by separately diluting the plasmid DNA in Opti-MEM reduced serum medium and diluting Lipofectamine 3000 reagent in Opti-MEM without serum or antibiotics, followed by mixing the DNA and Lipofectamine 3000 reagent at an appropriate ratio (typically 1–2 µg DNA per 1–2 µL reagent) and incubating at room temperature for 5–10 min to allow complex formation. The DNA-lipid complex was then added directly to the cells, gently swirled for even distribution, and incubated at 37 ℃ in a 5% CO_2_ incubator for 4–6 h. After incubation, the transfection mixture was replaced with complete growth medium containing serum and antibiotics, and cells were further cultured for 24–48 h to allow plasmid expression.

### Cell proliferation, cell cycle, and apoptosis assays

To evaluate cell proliferation, both colony formation and EdU incorporation assays were performed. For colony formation, cells were trypsinized, counted, and seeded into six-well plates at a density of 800–1000 cells per well. After 10–14 days of incubation with medium changes every 3–4 days, colonies were fixed in methanol for 20 min, stained with 0.5% crystal violet for 30 min, and photographed. Colonies consisting of ≥ 50 cells were counted manually.

For the EdU assay, cells were seeded in 96-well plates and incubated until they reached 80%–90% confluence. Cells were then exposed to 10 μmol/L EdU (Cat. #C0075S, Beyotime, Shanghai, China) for 2 h. After incubation, cells were fixed in 4% paraformaldehyde (PFA), permeabilized with 0.5% Triton X-100, and incubated with the click reaction cocktail according to the manufacturer's instructions. Nuclei were counterstained with Hoechst 33342, and positive cells were visualized and quantified using a high-content imaging system.

Cell cycle analysis was performed using flow cytometry. Cells were harvested, washed with phosphate-buffered saline (PBS; Cat. #G4202, Servicebio, Wuhan, China), and fixed in cold 70% ethanol at −20 ℃ overnight. Following fixation, cells were washed again and incubated with propidium iodide (PI) staining solution containing RNase A (Cat. #C1731, Beyotime) for 15 min at room temperature in the dark. DNA content was analyzed using a FACSCalibur flow cytometer (BD Biosciences, San Jose, CA, USA), and the distribution of cells in different cell cycle phases was calculated using FlowJo software.

Apoptosis was measured using an Annexin Ⅴ-FITC/PI apoptosis detection kit (Cat. #V13242, Invitrogen). Cells were collected, washed with cold PBS, and resuspended in binding buffer. FITC-conjugated Annexin Ⅴ and PI were added to the suspension and incubated for 15 min at room temperature in the dark. Samples were filtered through a 40-µm cell strainer to obtain a single-cell suspension and prevent clogging of the flow cytometer nozzle. After filtration, samples were immediately analyzed by flow cytometry to ensure viability and data accuracy. The percentage of early and late apoptotic cells was determined based on quadrant distribution.

### RNA pull-down and mass spectrometry

To identify proteins interacting with piR-61298, an RNA pull-down assay was performed using a biotin-labeled piR-61298 probe and a magnetic RNA-protein pull-down kit (Cat. #20164, Thermo Fisher Scientific). Briefly, 50 μL of streptavidin-coated magnetic beads were washed and incubated with 2 μg of biotinylated RNA at 4 ℃ overnight. After RNA coupling, beads were incubated with 100 μL of cell lysate containing RNase inhibitor for protein capture. Input samples were collected and stored at −80 ℃ for comparison. Following incubation, beads were thoroughly washed, and bound proteins were eluted by boiling in SDS buffer. A portion of the eluate was separated by SDS-PAGE and visualized by silver staining. Specific bands were excised and analyzed by liquid chromatography-tandem mass spectrometry (Genecreate, Wuhan, China). These bands were selected based on their differential expression patterns between experimental groups, characteristic molecular weights, or specific post-translational modifications. Bands excluded from analysis often consisted of staining artifacts, keratin contaminants, overloaded regions, or molecular weight markers, as these can interfere with peptide detection. Mass spectra were searched against the human protein database to identify candidate RNA-binding proteins. Selected proteins of interest were further validated by Western blotting (WB) following standard protocols.

### Co-immunoprecipitation (Co-IP)

Co-IP assays were performed using a magnetic bead-based kit (Cat. #88804, Thermo Fisher Scientific) to investigate protein-protein interactions. Briefly, cells were lysed with pre-cooled IP lysis buffer containing protease inhibitors and incubated on ice for 5–10 min. After centrifugation at 14000 *g* at 4 ℃ for 10 min, the supernatants were collected. For each reaction, 100 μg of total protein was incubated with 5 μg of specific antibodies against USP10 (Cat. #19374-1-AP, Proteintech, Wuhan, China) or p53 (Cat. #10442-1-AP, Proteintech) at 4 ℃ with gentle rotation overnight. An IgG antibody (Thermo Fisher Scientific) was used as a negative control. Protein A/G magnetic beads (25 μL) were pre-washed and then added to the antibody-protein mixtures and incubated at room temperature for 1 h. After washing the beads three times to remove nonspecific binding, the immunocomplexes were eluted in loading buffer and subjected to SDS-PAGE and WB for the detection of interacting proteins.

### RNA immunoprecipitation (RIP) assays

RIP assays were performed using the Magna RIP RNA-Binding Protein Immunoprecipitation Kit (Millipore, Burlington, MA, USA) to investigate the interaction between piR-61298 and USP10. HCT116 and LoVo cells were lysed, and the supernatants were incubated with magnetic beads conjugated to either anti-USP10 antibody (1∶50 dilution; Cat. #19374-1-AP, Proteintech) or normal IgG control (1∶50 dilution; Cat. #ab150075, Abcam, Cambridge, UK) at 4 ℃ with gentle rotation overnight. The beads were then washed thoroughly with RIP wash buffer to remove non-specific binding. RNA was extracted from the immunoprecipitates through proteinase K digestion followed by purification. The recovered RNA was reverse transcribed and analyzed by RT-qPCR to quantify piR-61298 enrichment. *GAPDH* or *U6* was used as the internal control. All experiments were performed in triplicate to ensure reproducibility.

### Fluorescence *in situ* hybridization (FISH) and immunofluorescence (IF) co-staining

FISH and IF co-staining were performed to examine the localization of piR-61298 and its colocalization with USP10 in CRC cells. A 5-carboxy-fluorescein (5-FAM)-labeled probe specific to piR-61298 (GenePharma, Shanghai, China) was used for RNA-FISH, with the probe sequences listed in ***Supplementary Table 6***. The hybridization was conducted using a Fluorescent *In Situ* Hybridization Kit (FISH Kit; Cat. #C10910, RiboBio). For colocalization analysis, cells were incubated overnight at 4 ℃ with an antibody against USP10 (1∶100; Cat. #19374-1-AP, Proteintech), followed by incubation with Cy3-conjugated secondary antibodies at 37 ℃ for 1 h. Following IF staining, RNA-FISH was performed using a 5-FAM-labeled piR-61298-specific probe according to the manufacturer's instructions provided with the FISH Kit. Finally, nuclei were counterstained using DAPI (Cat. #C1006, Beyotime).

### Nuclear-cytoplasmic co-localization

Cells were seeded onto sterile glass coverslips and cultured until approximately 70% confluence. The cells were then fixed with 4% PFA in PBS for 15 min at room temperature. After fixation, the cells were permeabilized with 0.3% Triton X-100 in PBS for 10 min to allow for probe penetration. Hybridization was carried out using a locked nucleic acid (LNA) probe (GenePharma) in a hybridization buffer, followed by incubation at 37 ℃ overnight in a humidified chamber. Post-hybridization washes were performed to eliminate any unbound probes. For IF staining, cells were blocked with 5% bovine serum albumin (BSA) and stained for F-actin. Nuclei were counterstained with DAPI to visualize the cell nuclei.

### WB analysis

Protein lysates were quantified, separated by SDS-PAGE, and transferred onto polyvinylidene difluoride (PVDF) membranes (Cat. #ISEQ00010, Millipore, Burlington, MA, USA). The membranes were blocked with 5% BSA at room temperature for 2 h, followed by an overnight incubation at 4 ℃ with the following primary antibodies (all at a 1∶1000 dilution): anti-USP10 (Cat. #19374-1-AP), anti-p53 (Cat. #10442-1-AP), and anti-p21 (Cat. #10355-1-AP) from Proteintech; and anti-GAPDH (Cat. #AF0006) from Beyotime. After thorough washing with TBST, the membranes were incubated with HRP-conjugated goat anti-rabbit IgG (H+L) (1∶2000; Cat. #SA00001-2,Proteintech) and HRP-conjugated goat anti-mouse IgG (H+L) (1∶1000; Cat. #SA00001-1, Proteintech) for 1 h. GAPDH was used as the loading control.

### Animal experiments

To investigate the effect of piR-61298 on tumor growth, male BALB/c nude mice (4–6 weeks old) were randomly assigned to two groups: the NC group and the piR-61298 overexpression group. Each group received an injection of 1 × 10^7^ HCT116 cells, either overexpressing piR-61298 or stably expressing NC, into the flanks of the mice. Tumor dimensions were measured every 2 days. All animal procedures were approved by the Animal Care and Use Committee of Nanjing Medical University (Approval No. IACUC-2007044).

### Statistical analysis

Statistical analyses were performed to determine group differences using appropriate tests (*e.g.*, Student's *t*-test and chi-square test) based on the type and distribution of the data. For survival analysis, the Kaplan–Meier method was used to estimate overall survival differences. To evaluate the prognostic significance of USP10 expression, Kaplan–Meier survival curves were generated using integrated CRC datasets from the TCGA database, and survival probabilities between subgroups were compared using the log-rank test. Comparisons of quantitative data with nonparametric distributions were performed using the Mann–Whitney *U* test for two-group comparisons or the Kruskal–Wallis test for multiple-group comparisons, followed by pairwise Mann–Whitney *U* tests with Bonferroni correction when appropriate. All statistical analyses were conducted using R software (version 4.1.3). Data were presented as the mean ± standard deviation. *P* values < 0.05 were considered statistically significant.

## Results

### piR-61298 was upregulated in CRC tissues

**Figure 1:**
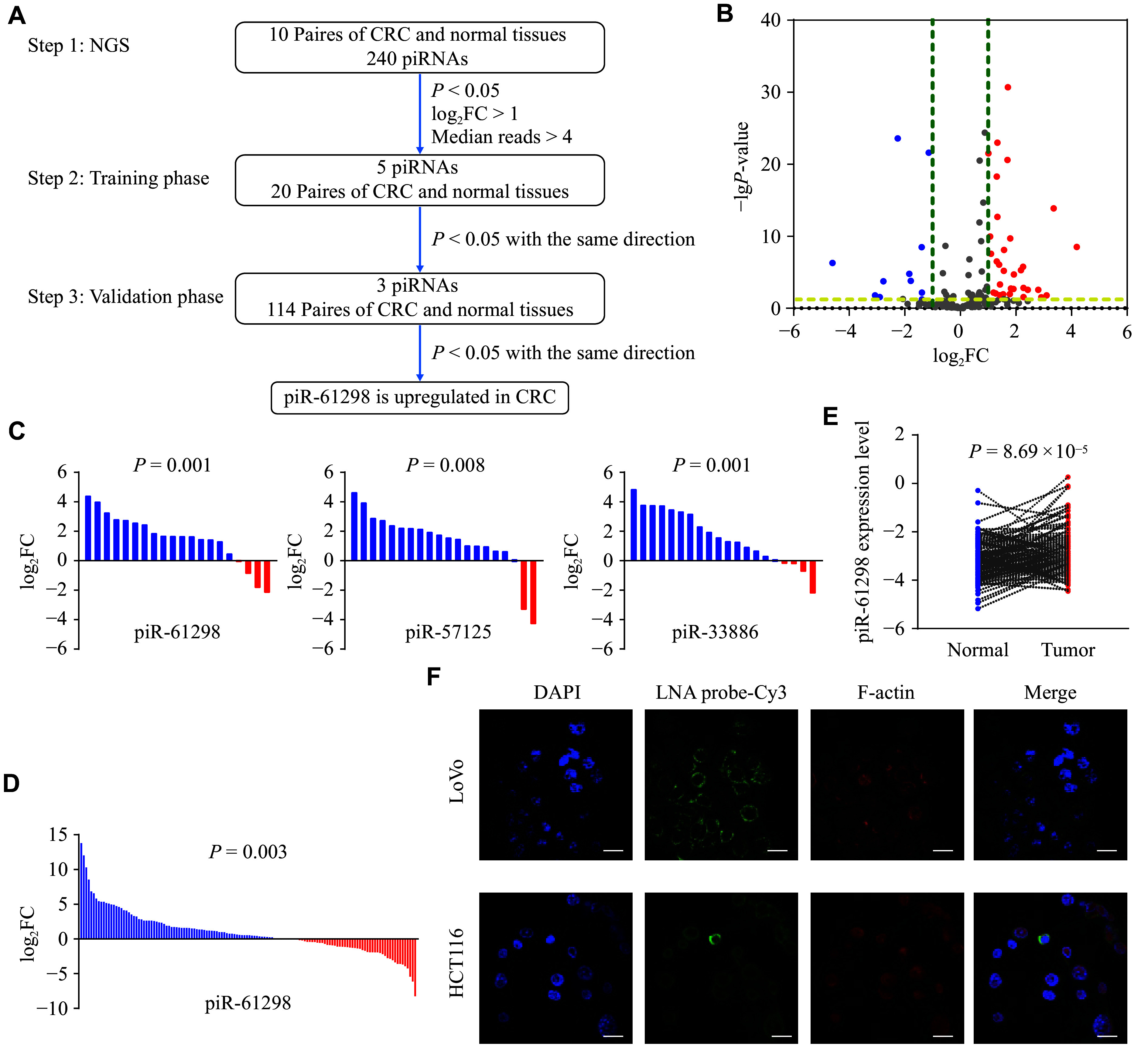
Colorectal cancer (CRC)-associated piRNA identification and expression analyses. A: Flow chart of piRNA screening. B: Volcano plot analysis of differential piRNAs in CRC and adjacent tissues identified by next-generation sequencing. C and D: The screened differential piRNAs were validated in expanded samples (C: initial validation samples, *n* = 20; D: expanded validation samples, *n* = 114). E: piR-61298 expression levels in CRC and adjacent tissues (*n* = 134). F: Fluorescence *in situ* hybridization using a locked nucleic acid (LNA) probe was performed to detect piR-61298 in CRC cells. Scale bar, 10 μm. Statistical analyses for panels C and D were performed using the Wilcoxon signed-rank test. Statistical analysis for panel E was performed using paired Student’s *t*-tests.

The workflow for identifying CRC-associated piRNAs is shown in ***Fig. 1A***. RNA extracted from 10 paired CRC and adjacent tissues was sequenced by NGS, identifying 240 piRNAs. Based on screening criteria including *P* < 0.05, |log_2_(fold change)| > 1, and median read count > 4, we identified five piRNAs meeting these criteria (***Fig. 1B***). To further validate these findings, we analyzed these piRNAs in 20 additional pairs of CRC and adjacent tissues, and identified three piRNAs with distinct characteristics (***Fig. 1C***). In the second phase, 114 pairs of CRC and adjacent tissues were included, and we found that only piR-61298 showed consistent differential expression in the same direction (***Fig. 1D***). piR-61298 expression was significantly elevated in CRC tissues compared with adjacent tissues (*P* = 8.69 × 10^−5^; ***Fig. 1E***). The association between piR-61298 expression and clinical characteristics of CRC is summarized in ***Supplementary Table 2***.

### piR-61298 promoted the malignant phenotypes of CRC cells *in vitro* and *in vivo*

To investigate the role of piR-61298 in CRC, we first analyzed its expression in various human CRC cell lines, as well as in a normal human colon mucosal epithelial cell line. The expression levels of piR-61298 were significantly higher in tumor cells than in normal cells (***Supplementary Fig. 1A***). Additionally, nucleocytoplasmic separation and LNA-FISH assays revealed that piR-61298 was predominantly localized in the cytoplasm (***Supplementary Fig. 1B*** and ***Fig. 1F***).

**Figure 2:**
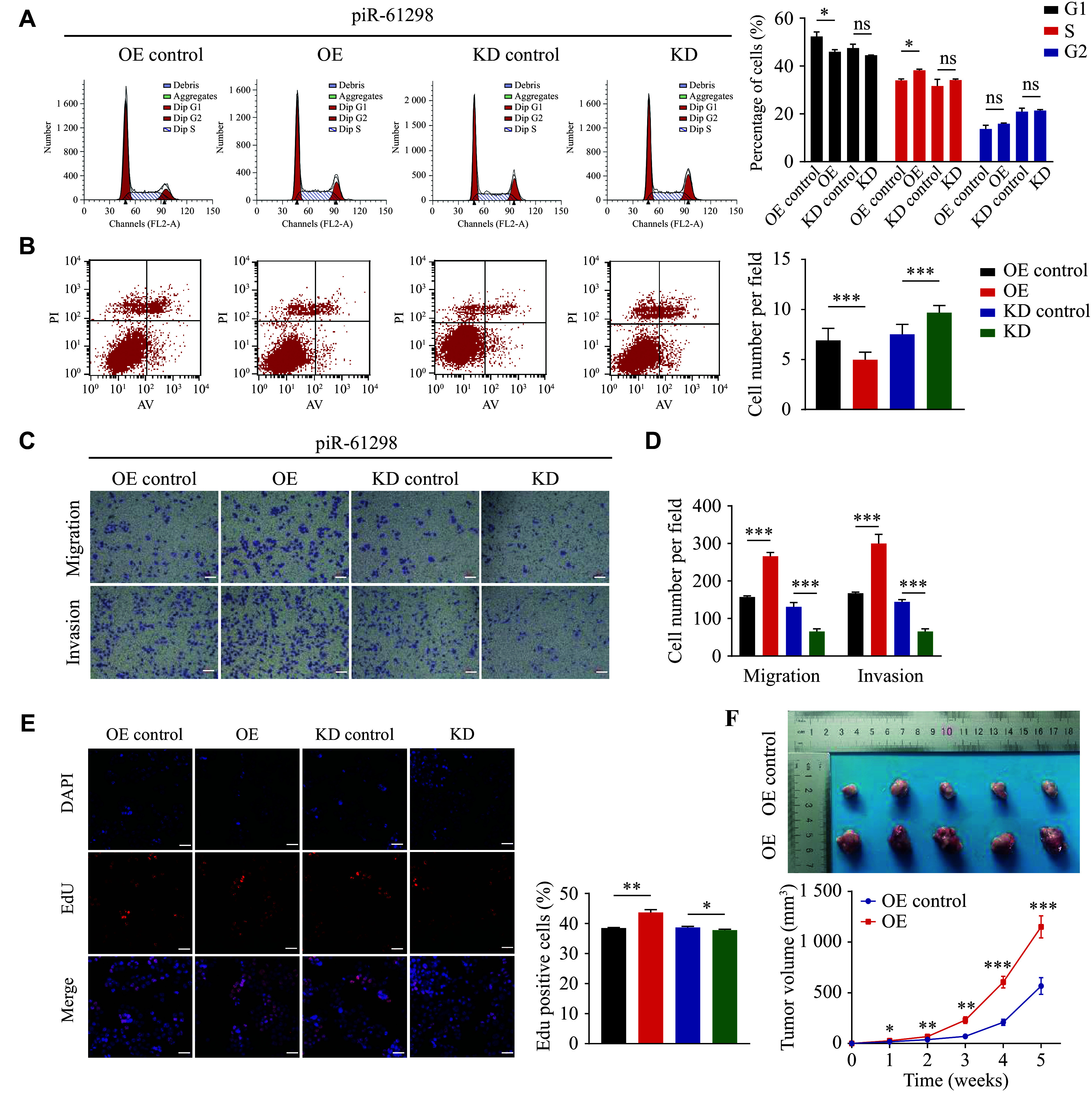
piR-61298 acted as an oncogene and promoted the malignant phenotype of colorectal cancer. A and B: Flow cytometry analysis of cell cycle distribution (A) and apoptosis (B) in HCT116 cells with or without piR-61298 overexpression (OE) or knockdown (KD). C and D: Analysis of migration and invasion abilities of HCT116 cells following piR-61298 overexpression or knockdown. Scale bar, 100 μm. E: Proliferation of HCT116 cells following piR-61298 overexpression or knockdown was measured by EdU assay. Scale bar, 100 μm. F: Representative images of xenograft tumors from nude mice injected with HCT116 cells overexpressing piR-61298 and control cells, and corresponding tumor growth curves (*n* = 5). Statistical analyses were performed using the Kruskal–Wallis test for multiple-group comparisons, followed by pairwise Mann–Whitney *U* tests with Bonferroni correction when appropriate. Data are presented as mean ± standard deviation; ^*^P < 0.05, ^**^P < 0.01, and ^***^P < 0.001.

To examine whether piR-61298 contributes to the malignant phenotype of CRC, we conducted a series of gain- and loss-of-function assays. Lentiviral vectors were used to establish stable piR-61298-overexpressing and piR-61298-knockdown cell lines in HCT116 and LoVo cells. The efficiency of piR-61298 overexpression and knockdown was confirmed by RT-qPCR (***Supplementary Fig. 2A***). Our results showed that piR-61298 overexpression promoted G1/S phase transition and accelerated cell cycle progression in CRC cells (***Fig. 2A***). Furthermore, it inhibited apoptosis (***Fig. 2B*** and ***Supplementary Fig. 2B***), enhanced cell invasion and migration (***Fig. 2C***, ***2D***, and ***Supplementary Fig. 2C***), promoted cell growth (***Fig. 2E*** and ***Supplementary Fig. 3A***), and increased colony formation (***Supplementary Fig. 3B***). In contrast, piR-61298 knockdown resulted in the opposite effects.

To assess the effect of piR-61298 on tumor growth *in* *vivo*, we established xenograft mouse models. The results showed that piR-61298 overexpression significantly promoted tumor growth, as indicated by an increased growth rate and larger tumor diameters (***Fig. 2F***). Taken together, these findings indicate that piR-61298 promotes CRC proliferation and metastasis, thereby contributing to CRC malignant progression.

### piR-61298 directly bound to the deubiquitinase USP10

**Figure 3:**
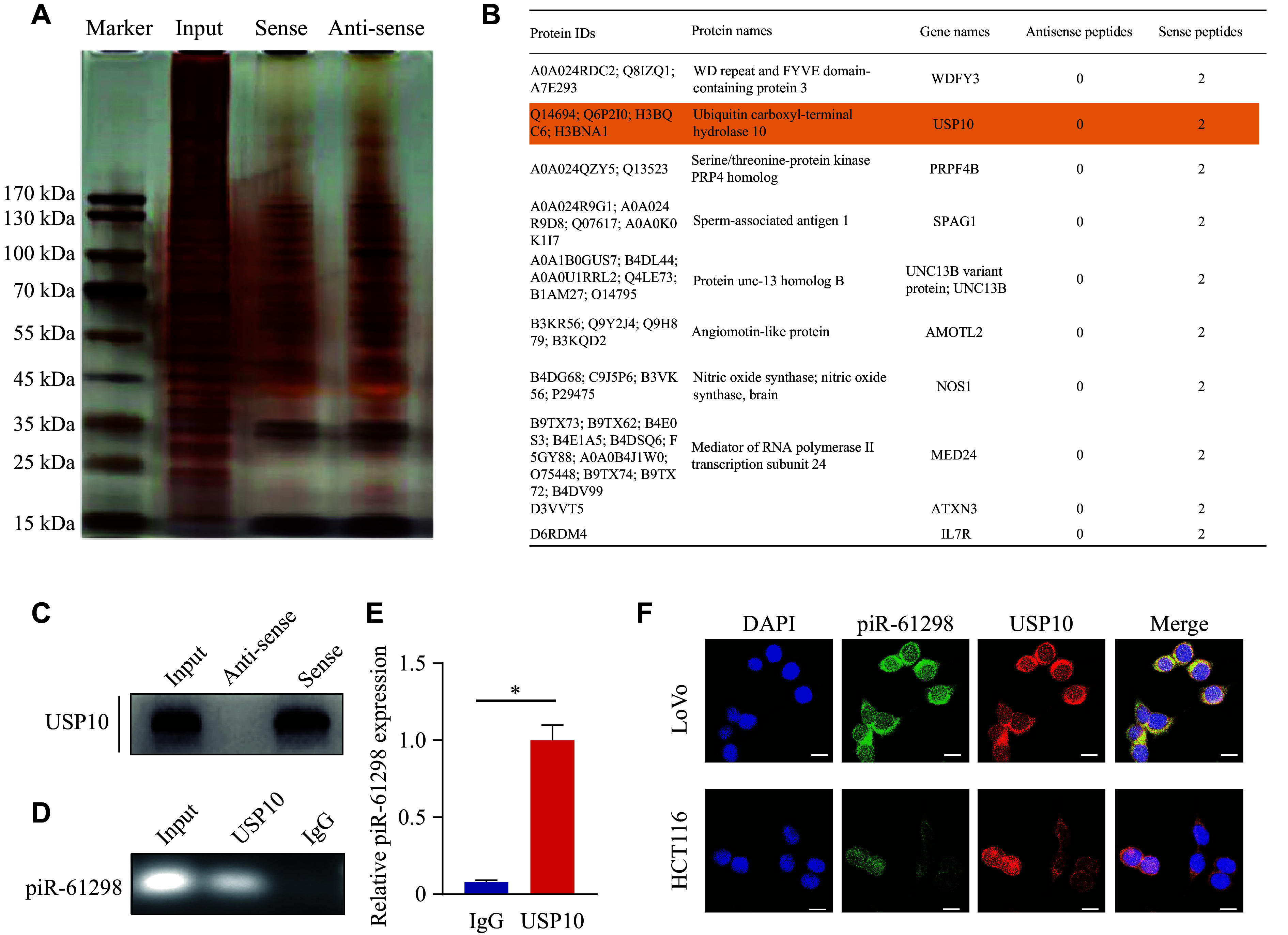
Identification of the interaction between USP10 protein and piR-61298. A: Protein extracts from colorectal cancer (CRC) cells were incubated with a biotin-labeled piR-61298 sense probe or antisense probe, and the piR-61298-protein complexes were visualized by silver staining. B: Identification of proteins binding to piR-61298 by mass spectrometry. C: Western blotting analysis of USP10 enriched by piR-61298 probes in CRC cells. D and E: RNA immunoprecipitation quantitative PCR analysis of piR-61298 immunoprecipitated in CRC cells using an anti-USP10 antibody and agarose assay. F: USP10 immunofluorescence (red) and fluorescence *in situ* hybridization analysis of piR-61298 (green) in HCT116 and LoVo cells. Scale bar, 10 μm. Nuclei were counterstained with DAPI (blue). Statistical analysis for panel E was performed using the Mann–Whitney *U* test. Data are presented as mean ± standard deviation (*n* = 3). ^*^P < 0.05. Abbreviation: USP10, ubiquitin-specific peptidase 10.

Given that piR-61298 promotes the malignant phenotype of CRC, we next investigated the underlying molecular mechanisms. piRNAs exert their functions by interacting with proteins, forming RNA-protein complexes that regulate various cellular processes. To identify the protein partners of piR-61298, we performed a biotin-streptavidin RNA pull-down assay using biotinylated piR-61298. Several proteins enriched in the piR-61298 pull-down fraction, compared with the antisense control, were analyzed by mass spectrometry (***Fig. 3A***). The results showed that piR-61298 specifically bound to the deubiquitinating enzyme USP10 and to PIWIL1 (***Fig. 3B*** and ***Supplementary Fig. 4***). Further validation experiments demonstrated that piR-61298 could pull down USP10 in CRC cells (***Fig. 3C*** and ***Supplementary Fig. 5***). The interaction between piR-61298 and USP10 was also confirmed by RIP followed by agarose gel electrophoresis (***Fig. 3D*** and ***3E***). Additionally, we observed that piR-61298 and USP10 were colocalized in the cytoplasm (***Fig. 3F***). These results suggest that piR-61298 may regulate cellular processes by interacting with USP10.

### USP10 interacted with p53 and was highly expressed in CRC

**Figure 4:**
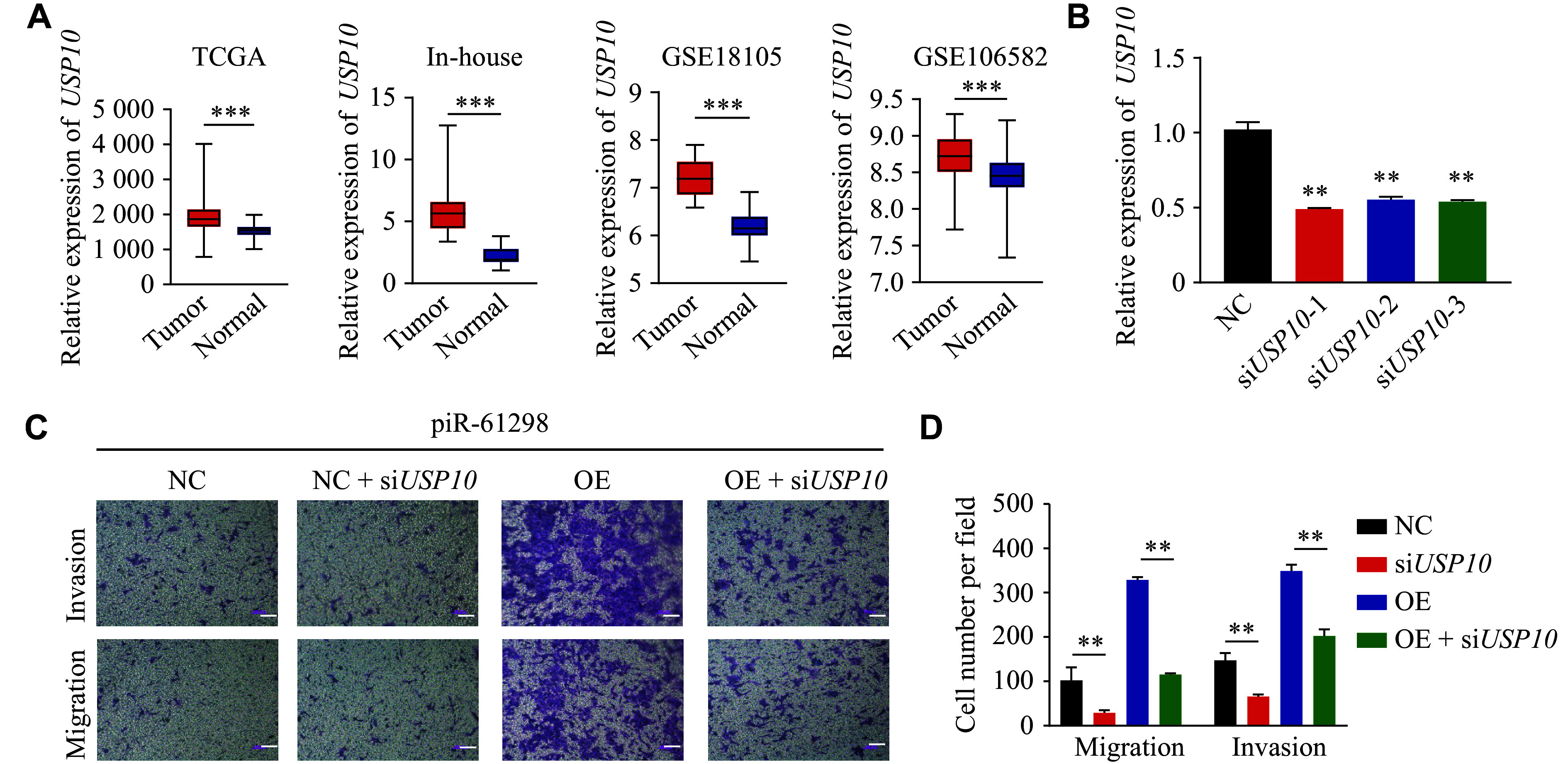
USP10 promoted the malignant progression of colorectal cancer (CRC). A: USP10 expression levels in CRC and normal tissues from TCGA, GEO, and in-house datasets. B: Verification of the knockdown efficiency of siRNAs targeting *USP10*. C and D: Effects of *USP10* knockdown (*via* si*USP10*) on the migration and invasion abilities of HCT116 cells overexpressing (OE) piR-61298. Scale bar, 100 μm. For panel A, differences between tumor and normal tissues were analyzed using Student’s *t*-test or the Mann-Whitney *U* test, as appropriate. Statistical analyses for panels B–D were performed using the Kruskal–Wallis test for multiple-group comparisons, followed by pairwise Mann–Whitney *U* tests with Bonferroni correction when appropriate. Data are presented as mean ± standard deviation (*n* = 3). ^**^P < 0.01, and ^***^P < 0.001. Abbreviations: GEO, Gene Expression Omnibus; TCGA, The Cancer Genome Atlas; USP10, ubiquitin-specific peptidase 10.

Analysis of the TCGA, GSE18105, GSE106582, and our in-house datasets revealed that USP10 expression was consistently elevated in CRC tissues, compared with adjacent normal tissues (***Fig. 4A***). We also observed that PIWIL1, a known binding partner of piR-61298, was highly expressed in tumor samples (***Supplementary Fig. 6A***). However, when we analyzed the TCGA dataset for potential correlations between USP10 expression and patient prognosis, no statistically significant association was identified (***Supplementary Fig. 6B***). This lack of correlation may be attributed to the fact that mRNA expression levels do not always reflect functional protein activity or biological outcomes.

**Figure 5:**
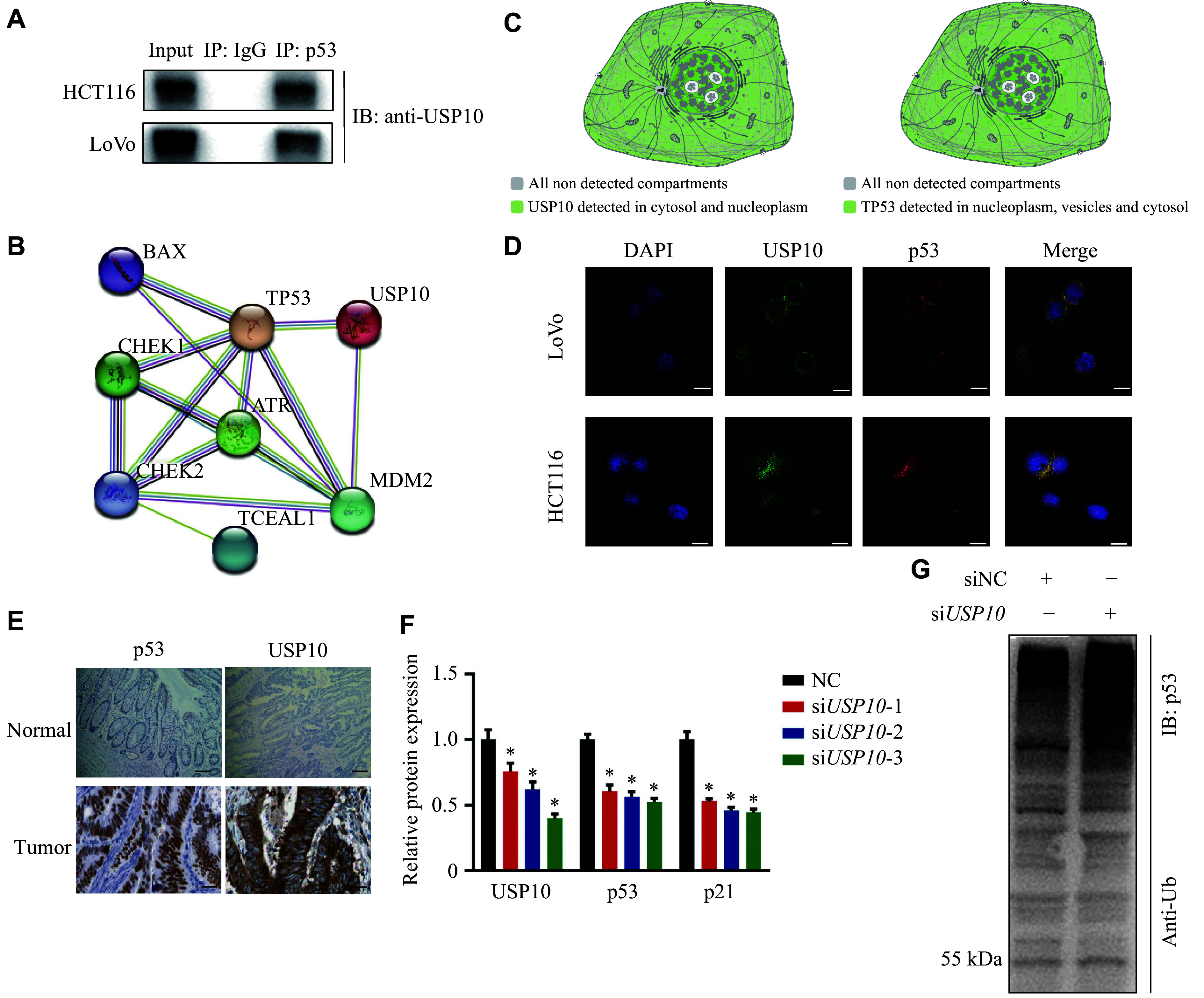
USP10 bound to p53 and stabilized its expression through deubiquitination. A: Co-IP assay using an anti-USP10 antibody was performed to detect the interaction between USP10 and p53. B: Analysis of the interaction between USP10 and p53 was predicted by the protein-protein interaction network. C: Subcellular localization of USP10 and p53 from the Human Protein Atlas (HPA) database. D: Co-localization analysis by immunofluorescence staining of USP10 and p53. Scale bar, 10 μm. E: Immunohistochemical staining of USP10 and p53 in normal and tumor tissues, obtained from the HPA database. Scale bar, 100 μm. F: Relative protein levels of p53 and p21 in HCT116 cells following treatment with si*USP10* for 24 h. G: Effect of *USP10* knockdown (*via* si*USP10*) on p53 ubiquitination in HCT116 cells. Statistical analysis for panel F was performed using the Kruskal–Wallis test, followed by pairwise Mann–Whitney *U* tests with Bonferroni correction when appropriate. Data are presented as mean ± standard deviation (*n* = 3). ^*^P < 0.05. Abbreviation: USP10, ubiquitin-specific peptidase 10.

To further investigate the functional role of USP10 in CRC, we transiently transfected *USP10*-specific siRNAs (si*USP10*) into HCT116 and LoVo cells and confirmed the knockdown efficiency by RT-qPCR (***Fig. 4B***). Functional assays revealed that silencing *USP10* partially suppressed the increased migration and invasion induced by piR-61298 overexpression (***Fig. 4C***, ***4D***, and ***Supplementary Fig. 7***), indicating that piR-61298 exerts its oncogenic effects, at least in part, through USP10. Moreover, Co-IP experiments demonstrated a physical interaction between USP10 and p53 in CRC cells (***Fig. 5A***), which was further supported by protein-protein interaction network analysis (***Fig. 5B***). Together, these results suggest that USP10 may promote CRC progression by interacting with p53 and modulating its downstream signaling pathways.

### USP10 negatively regulated p53 ubiquitination by deubiquitination

We further investigated the molecular mechanisms by which the interaction among piR-61298, USP10, and p53 promotes CRC progression. We found that USP10 and p53 colocalized in the cytoplasm at both the cellular and tissue levels (***Fig. 5C***–***5E***). Additionally, knockdown of *USP10* resulted in significantly reduced protein levels of both p53 and p21 (***Fig. 5F*** and ***Supplementary Fig. 8***). Moreover, Co-IP experiments revealed a significant increase in p53 ubiquitination following USP10 knockdown (***Fig. 5G***).

### piR-61298 promoted CRC cell growth by disrupting the USP10-p53 interaction

**Figure 6:**
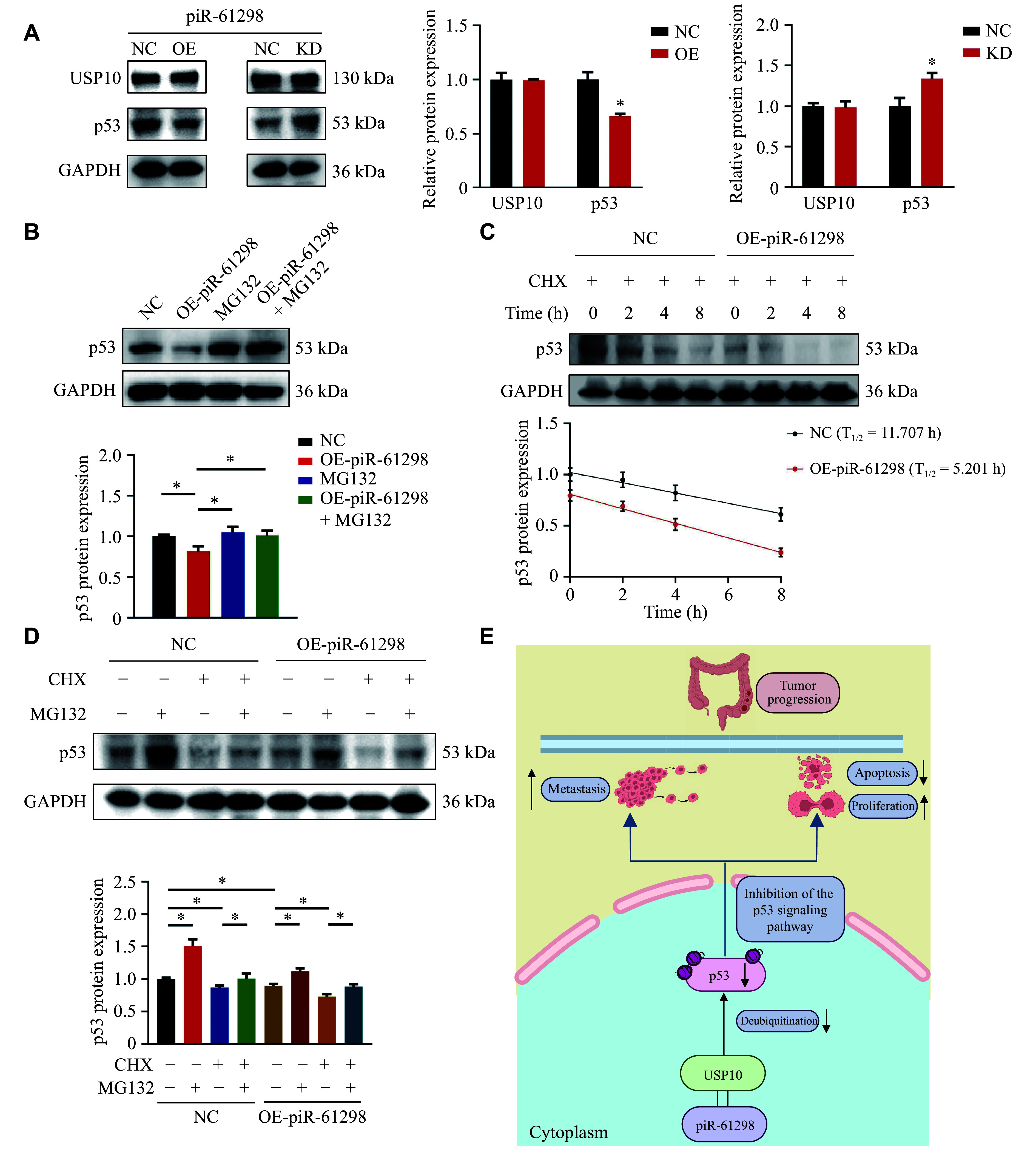
piR-61298 promoted colorectal cancer (CRC) cell growth by inhibiting the interaction between USP10 and p53. A: Western blotting (WB) analysis of p53 expression in HCT116 cells with piR-61298 overexpression (OE) or knockdown (KD). B: WB analysis of p53 protein expression following MG132 (10 µmol/L) treatment and piR-61298 overexpression. C: p53 protein levels in the control and piR-61298 overexpression groups at the indicated time points after CHX (50 µg/mL) treatment were detected by WB. D: p53 protein levels in the control and piR-61298 overexpression groups after cotreatment with CHX and MG132 were detected by WB. E: Proposed schematic model illustrating that piR-61298 promotes CRC progression by interacting with USP10 to regulate the p53 signaling pathway. Statistical analysis for panel A was performed using the Mann–Whitney *U* test. Statistical analyses for panels B and D were performed using the Kruskal–Wallis test for multiple-group comparisons, followed by pairwise Mann–Whitney *U* tests with Bonferroni correction when appropriate. Data are presented as mean ± standard deviation (*n* = 3). ^*^P < 0.05. Abbreviation: CHX, cycloheximide.

The above results prompted us to further investigate the role of piR-61298 in the USP10 and p53 signaling pathways. We examined the effect of piR-61298 on USP10 and p53 protein levels and found that piR-61298 overexpression significantly decreased p53 levels, whereas USP10 expression remained unaffected (***Fig. 6A***). This suggests that piR-61298 does not regulate USP10 expression, but may instead facilitate p53 ubiquitination by binding to USP10, thereby promoting CRC progression.

Next, we treated cells with MG132, a proteasomal inhibitor, and found that MG132 prevented the decrease in p53 levels caused by piR-61298 overexpression, indicating that piR-61298 overexpression reduces p53 levels by promoting its proteasomal degradation (***Fig. 6B***). We also treated cells with cycloheximide (CHX), an inhibitor of protein synthesis, and observed that p53 expression gradually decreased over time. Notably, piR-61298 overexpression significantly accelerated p53 degradation (***Fig. 6C***), an effect that was abolished by MG132 treatment (***Fig. 6D***). Given that the proteasome is the primary pathway for the degradation of ubiquitinated proteins, Co-IP results revealed that piR-61298 overexpression enhanced p53 ubiquitination by inhibiting the deubiquitination activity of USP10. This effect was reversed by USP10 overexpression (***Supplementary Fig. 9***).

## Discussion

In the present study, we identified a piRNA, piR-61298, which was significantly upregulated in CRC samples. Overexpression of piR-61298 enhanced the viability of CRC cells *in vitro*, promoting cell proliferation, migration, and invasion. Additionally, a nude mouse xenograft model demonstrated the tumor-promoting effect of piR-61298 *in vivo*. Our findings highlight the role of piR-61298 in CRC progression and suggest its potential as a promising biomarker for CRC.

piRNAs have been reported to influence cancer development by regulating gene expression and protein interactions^[16]^. To date, only a limited number of studies have explored the role of piRNAs in the development and progression of CRC. For example, piR-54265 binds to PIWIL2 and promotes CRC proliferation and metastasis through the STAT3 signaling pathway by modulating the PIWIL2/STAT3/p-SRC complex^[17]^. Another piRNA, piR-823, enhances cell proliferation, invasion, and apoptosis resistance in CRC cells by modulating the G6PD/HIF-1α pathway^[18]^. Additionally, piR-1245 has been shown to exhibit oncogenic properties and may serve as a potential prognostic biomarker for CRC^[19]^. Our study demonstrates that piR-61298 binds to USP10, thereby regulating the p53 signaling pathway and contributing to CRC progression.

USP10, a member of the USP family, is a deubiquitinating enzyme that has been shown to play a critical role in the progression of various cancers. It has been shown to localize in both the cytoplasm and nucleus of cells^[20]^. Our experiments further demonstrated that USP10 interacted with piR-61298 in the cytoplasm. Growing evidence supports the important role of USP10 in cancer progression. For instance, USP10 interacts with NLRP7 in CRC cells and catalyzes its deubiquitination, thereby promoting M2 macrophage polarization and contributing to CRC development and metastasis^[21]^. Additionally, USP10 can interact with melatonin, leading to the destabilization of HDAC7 protein and inhibition of cell growth to suppress esophageal squamous cell carcinoma progression^[22]^. Notably, WDFY3 was also identified with high specificity in the pull-down assay, suggesting a potential interaction with piR-61298. WDFY3 has been characterized as a scaffold protein involved in selective autophagy and protein aggregate clearance. While biologically important, especially in neuronal contexts, its direct involvement in tumorigenesis remains less well established. In contrast, USP10 is a deubiquitinating enzyme with a broad range of functions, many of which are highly relevant to cancer development, including regulation of p53, AMPK signaling, and DNA damage response^[23–24]^. These functional differences, particularly the enzymatic role of USP10 and the extensive literature on its involvement in cancer, justify its prioritization for further validation in the present study.

The biological functions and underlying mechanisms of USP10 in CRC remain poorly understood. The specific role of USP10 in tumorigenesis is influenced by its interaction with substrates^[15,25]^. Previous studies have shown that USP10 binds to p53 and contributes to tumor development^[26]^. p53, a well-established tumor suppressor, plays a crucial role in regulating the cell cycle by activating its target gene *P21*, which in turn inhibits cell proliferation^[27]^. Our findings suggest that piR-61298 downregulates p53 protein levels, and that USP10 modulates p21, a downstream target of p53. Moreover, our research supports the notion that USP10 mediates the deubiquitination of p53, regulating its activity in the cytoplasm. Additional studies have provided evidence that circWSB1 interacts with USP10 and disrupts its binding to p53^[28]^, leading to the polyubiquitination and degradation of p53, thereby promoting breast cancer progression^[29]^. Similarly, the interaction between USP10 and G3BP2 not only inhibits p53 signaling but also facilitates prostate cancer progression^[30]^. Further *in vitro* and *in vivo* validation demonstrated that piR-61298 may promote CRC progression by destabilizing p53 through its interaction with USP10.

Although both circWSB1 and piR-61298 influence the USP10/p53 axis by modulating the deubiquitinating activity of USP10, they represent mechanistically and structurally distinct types of non-coding RNAs. circWSB1 is a covalently closed circular RNA that primarily functions as a molecular scaffold or decoy, facilitating or disrupting protein-protein interactions. In contrast, piRNAs are small single-stranded RNAs (24–32 nt) that canonically associate with PIWI proteins and are best known for their roles in transposon silencing and epigenetic regulation in the germline^[31]^. Recent studies, however, have suggested that piRNAs can also exert non-canonical functions in somatic cells, including direct interaction with proteins independent of PIWI, potentially altering protein conformation, enzymatic activity, or subcellular localization^[32]^. In the current study, piR-61298 appeared to impair the capacity of USP10 to deubiquitinate p53 without affecting USP10 expression, suggesting a non-transcriptional, possibly direct post-translational regulatory mechanism. We acknowledge that the structural determinants and PIWI-independent binding interfaces of piR-61298 were not systematically explored in our current work. Nevertheless, prior studies have indicated that certain piRNAs may harbor secondary structures or sequence motifs conducive to direct protein interaction, offering a potential mechanistic basis for their observed effects^[33]^. This implies that piRNAs may modulate protein function through interaction surfaces or structural disruption in a manner distinct from the scaffold-like roles commonly attributed to circRNAs. These functional differences, rooted in their distinct biogenesis and molecular features, suggest that piRNAs represent a unique layer of post-translational regulation in cancer and warrant further mechanistic investigation.

USP10 is a well-characterized deubiquitinase that stabilizes p53 by removing polyubiquitin chains and preventing its degradation *via* the proteasome. Several studies have shown that USP10 overexpression is sufficient to reverse stress-induced p53 degradation^[34]^. Additionally, USP10 is known to translocate to the nucleus in response to cellular stress, enhancing p53 stability and promoting its tumor-suppressive functions^[35]^. In the current study, exogenous expression of USP10 in piR-61298-overexpressing CRC cells partially reversed the reduction of p53 protein levels. This observation is consistent with the hypothesis that piR-61298 impairs the function of USP10. However, additional validation, including the use of USP10 catalytic mutants and further mechanistic studies, is needed and will be addressed in future work.

Although we did not directly measure the expression of canonical p53 transcriptional targets such as PUMA and BAX, the observed reduction in p53 protein levels in piR-61298-overexpressing CRC cells strongly implies impairment of its downstream regulatory network. As a central tumor suppressor, p53 exerts its function by transactivating a wide array of genes involved in apoptosis (*e.g.*, *BAX*, *PUMA*, and *NOXA*), cell cycle arrest (*e.g.*, *CDKN1A*), and DNA repair (*e.g.*, *GADD45A *and *DDB2*)^[36]^. In CRC, p53-mediated induction of mitochondrial apoptotic effectors such as PUMA and BAX is essential for eliminating cells with oncogenic mutations or genotoxic stress. Simultaneously, activation of CDKN1A halts G1/S progression to allow DNA repair, while GADD45A and DDB2 participate in genomic maintenance. Loss of p53 function or degradation, therefore, compromises these coordinated protective responses and contributes to malignant transformation. Prior studies have demonstrated that p53 inactivation leads to apoptosis evasion, uncontrolled cell proliferation, and genomic instability, which are hallmarks of tumor progression^[37]^. In this context, the downregulation of p53 protein by piR-61298 strongly suggests a broader suppression of tumor suppressor pathways, even though downstream effectors were not directly assessed.

The unique biological characteristics of piRNAs render them promising candidates for therapeutic applications. Compared with miRNAs or siRNAs, piRNAs exhibit superior chemical stability, resistance to exonuclease-mediated degradation, and the ability to regulate gene expression through both PIWI-dependent and PIWI-independent mechanisms, including direct protein interactions^[7]^. These features, combined with their tissue-specific expression and sequence selectivity, make piRNAs attractive targets for precision intervention. In the current study, piR-61298 was found to impair USP10-mediated p53 deubiquitination without altering USP10 expression, suggesting a possible non-canonical regulatory mechanism through direct protein binding. Although piRNA-targeted therapies remain in the early stages of exploration, emerging studies have begun to adapt principles from miRNA inhibition strategies, such as the use of chemically modified antisense oligonucleotides (ASOs), to modulate piRNA function. For instance, LNA-based ASOs, which have demonstrated efficacy in miRNA-targeted preclinical and clinical trials, could theoretically be adapted to suppress oncogenic piRNAs with high specificity and stability^[38]^. While we did not directly test this approach, our findings provide a mechanistic rationale to explore such strategies in tumors with aberrant piR-61298 expression.

In the present study, we identified a piRNA, piR-61298, which was significantly differentially expressed between CRC and normal tissues. Further analysis demonstrated that piR-61298 bound to USP10, thereby promoting p53 ubiquitination and degradation. This molecular mechanism may contribute to the development of CRC. Our findings highlight the potential of piR-61298 as a therapeutic target for CRC.

## Additional information

The online version contains supplementary material available at http://www.jbr-pub.org.cn/article/doi/10.7555/JBR.39.20250137?pageType=en.
