# Supplementary material for: piR-61298 promotes colorectal cancer progression through destabilizing p53 by interacting with USP10
Source: J Biomed Res. 2026 May 21;40(3):266–79. doi: 10.7555/JBR.39.20250137 (PMC13231362; doi:10.7555/JBR.39.20250137)
Supplement: Supplementary file 1 — The online version contains supplementary material available at http://www.jbr-pub.org.cn/article/doi/10.7555/JBR.39.20250137?pageType=en. [file jbr-40-3-266-S1.pdf]

Available online at www.jbr-pub.org.cn

Open Access at PubMed Central

Journal of Biomedical Research, 2026 40(3): 1–6

Supplementary Data

JBR

piR-61298 promotes colorectal cancer progression through destabilizing p53 by interacting with USP10

Shenya Xu[1,2,3,△], Zhutao Ding[1,2,△], Shuai Ben[2], Chen Li[1], Silu Chen[1,2], Lingyan Zhao[1,2], Shuwei Li[2,#,✉], Dongying Gu[1,#,✉]

[1]*Department of Oncology, Nanjing First Hospital, Nanjing Medical University, Nanjing, Jiangsu 210006, China;*
[2]*Department of Genetic Toxicology, The Key Laboratory of Modern Toxicology of Ministry of Education, Key Laboratory of Public Health Safety and Emergency Prevention and Control Technology of Higher Education Institutions in Jiangsu Province, Center for Global Health, School of Public Health, Nanjing Medical University, Nanjing, Jiangsu 211166, China;*
[3]*Key Laboratory of Environmental Medicine Engineering, Ministry of Education, School of Public Health, Southeast University, Nanjing, Jiangsu 211189, China.*

Supplementary Table 1 **Demographic and clinical characteristics of the colorectal cancer cohort**

Clinical features	Training phase	Validation phase	P^a
Total	20	114	
Age (years)			0.959
≤ 60	9	52	
> 60	11	62	
Sex			0.246
Male	14	64	
Female	6	50	
Tumor site			0.190
Rectum	13	56	
Colon	7	58	
Tumor size (cm)			0.565
< 5.0	14	69	
≥ 5.0	5	34	
Missing data	1	11	
Tumor grade			0.908[a]
Low	0	1	
Intermediate	16	89	
High	4	24	

[△]These authors contributed equally to this work.
[#]These authors jointly supervised this work.

[✉]Corresponding authors: Shuwei Li, E-mail: shuweili@njmu.edu.cn, ORCID: 0000-0002-6142-0573; Dongying Gu, E-mail: dygu@njmu.edu.cn, ORCID: 0000-0002-5831-6775.

Received: 02 April 2025; Revised: 18 June 2025; Accepted: 24 June 2025; Available online: 22 July 2025; Published date: 21 May 2026

CLC number: R735.34, Document code: A

The authors reported no conflict of interests.

This is an open access article under the Creative Commons Attribution (CC BY 4.0) license, which permits others to distribute, remix, adapt and build upon this work, for commercial use, provided the original work is properly cited.

© 2026 by Journal of Biomedical Research.

https://doi.org/10.7555/JBR.39.20250137

Supplementary Table 1 **Demographic and clinical characteristics of the colorectal cancer cohort** (continued)

Clinical features	Training phase	Validation phase	P[a]
Dukes stage			0.562
A	1	4	
B	14	63	
C	5	45	
D	0	2	

[a]*P*-values were calculated with Fisher's exact test.

Supplementary Table 2 **Association between piR-61298 status and clinical characteristics of colorectal cancer**

Variables	Low piR-61298		High piR-61298		P[a]
	n	%	*n*	%	
Total	67		67		
Age (years)					0.862
≤ 60	30	44.8	31	46.3	
> 60	37	55.2	36	53.7	
Sex					0.484
Male	41	61.2	37	55.2	
Female	26	38.8	30	44.8	
Tumor site					0.863
Rectum	34	50.7	35	52.2	
Colon	33	49.3	32	47.8	
Tumor size (cm)[b]					0.174
< 5.0	38	56.7	45	67.2	
≥ 5.0	23	34.3	16	23.9	
Tumor grade					0.671
Low/intermediate	52	77.6	54	80.6	
High	15	22.4	13	19.4	
Dukes stage					1.000
A/B	41	61.2	41	61.2	
C/D	26	38.8	26	38.8	

[a]Two-sided χ^2-test for the frequency distribution of selected variables between high expression and low expression of piR-61298 in colorectal cancer.

[b]Tumor size data were missing for six patients in each group.

Supplementary Table 3 **Primer sequences for RT-qPCR**

Genes	Primers	Sequences (5′-3′)
USP10	Forward	ATTGAGTTTGGTGTCGATGAAGT
	Reverse	GGAGCCATAGCTTGCTTCTTTAG
piR-61298	Forward	TTGCTGTGATGACTATCTTAGGACA
	Reverse	AGTGCAGGGTCCGAGGTATT
GAPDH	Forward	CCGGGAAACTGTGGCGTGATGG
	Reverse	AGGTGGAGGAGTGGGTGTCGCTGTT

Supplementary Table 4 **siRNA sequences**

siRNA	Sequences (5′-3′)
si*USP10*-1	GGACAAGAAUAUCAGAGAATT
si*USP10*-2	GCUUUGGAUGGAAGUUCUATT
si*USP10*-3	GCAGGUUGAAGUCAAAGAATT

Supplementary Table 5 **The sequence of the antagomir**

piRNA	Sequences (5′-3′)
piR-61298	CAAAGGUGUCCUAAGAUAGUCAUCA CAGCAA

Supplementary Table 6 **Fluorescence *in situ* hybridization sequences**

piRNA	Sequences (5′-3′)
piR-61298	dig-CAAAGGTGTCCTAAGATAGTC ATCACAGCAA-dig

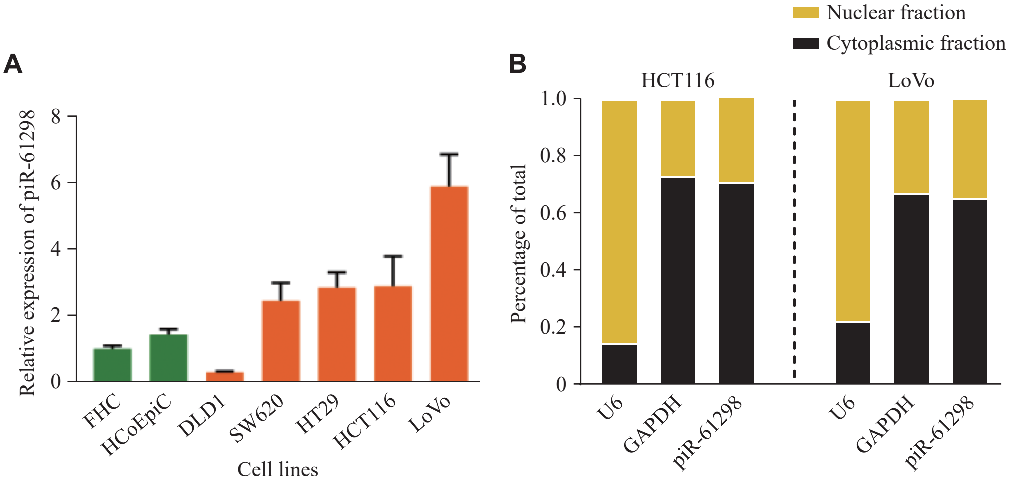

Supplementary Fig. 1 **Expression and subcellular localization of piR-61298.** A: Relative expression levels of piR-61298 in colorectal normal epithelial cells (green) and tumor cells (red). B: Distribution of piR-61298 in the nucleus and cytoplasm of colorectal cancer cells, using *U6* and *GAPDH* as nuclear and cytoplasmic markers, respectively.

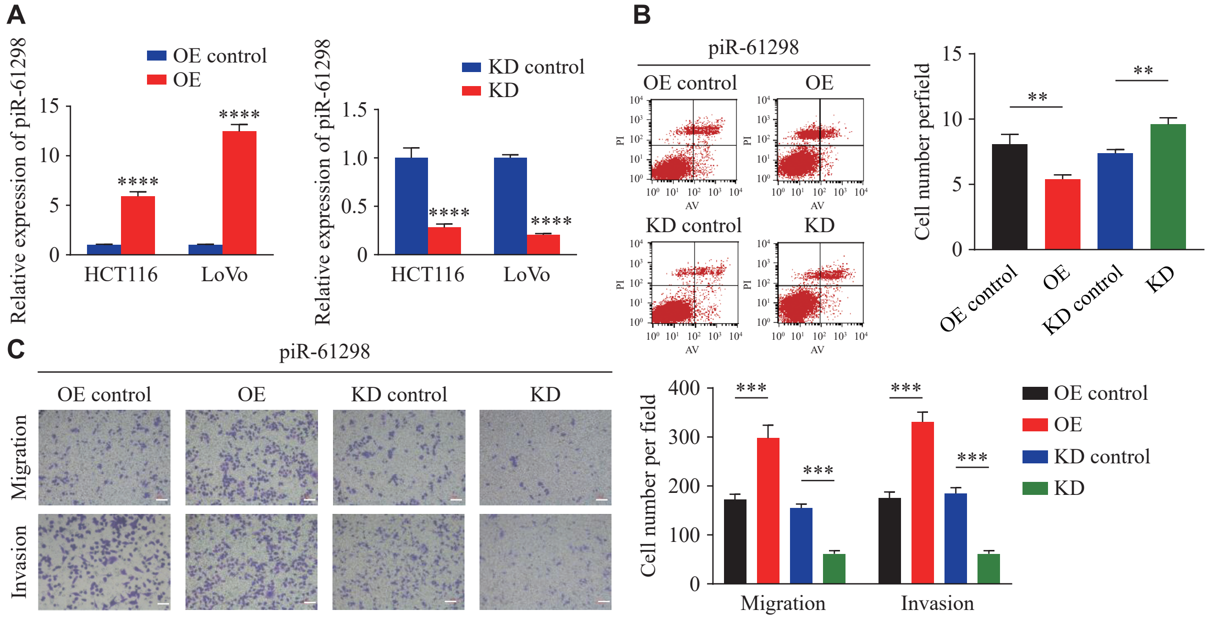

Supplementary Fig. 2 **Identification of the oncogenic role of piR-61298.** A: Efficiency of the constructed piR-61298 lentivirus. B: Effect of piR-61298 overexpression (OE) or knockdown (KD) on apoptosis in LoVo cells. C: Effect of piR-61298 overexpression (OE) or knockdown (KD) on the migration and invasion abilities of LoVo cells. Scale bar, 100 μm. Statistical analyses were performed using the Kruskal–Wallis test for multiple-group comparisons, followed by pairwise Mann–Whitney *U* tests with Bonferroni correction when appropriate. Data are presented as mean ± standard deviation ($n = 3$); $^{**}P < 0.01$, $^{***}P < 0.001$, and $^{****}P < 0.0001$.

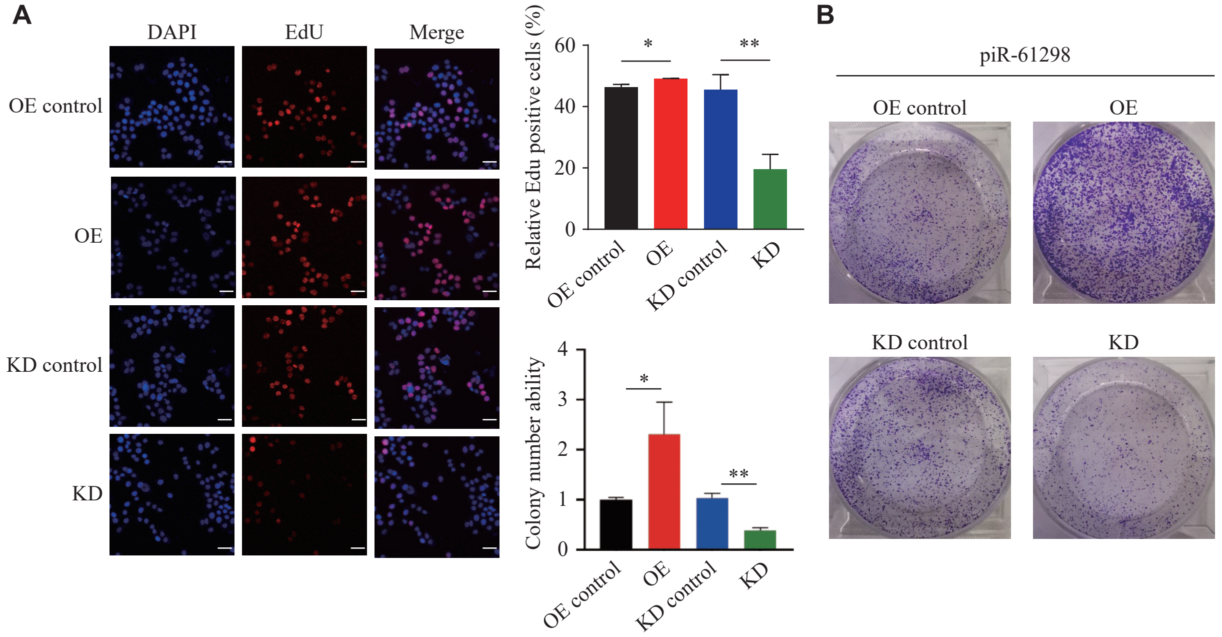

Supplementary Fig. 3 **piR-61298 promoted colorectal cancer cell proliferation.** A: The effects of piR-61298 overexpression (OE) or knockdown (KD) on the proliferation of LoVo cells were tested by EdU assay. Scale bar, 100 μm. B: The effects of piR-61298 overexpression (OE) or knockdown (KD) on the colony formation ability of LoVo cells were examined by colony formation assay. Statistical analyses were performed using the Kruskal–Wallis test for multiple-group comparisons, followed by pairwise Mann–Whitney U tests with Bonferroni correction when appropriate. Data are presented as mean ± standard deviation (n = 3); $^{*}P < 0.05$, $^{**}P < 0.01$.

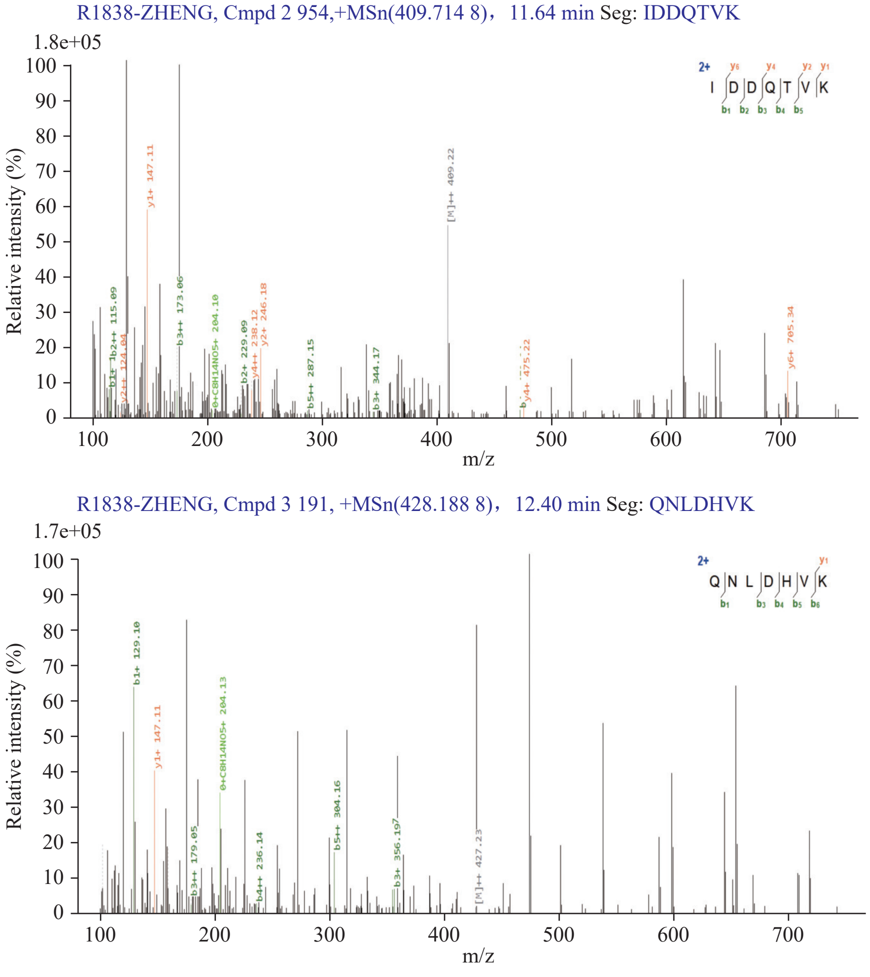

Supplementary Fig. 4 **Binding protein analysis of piR-61298.** Identification of binding proteins to piR-61298 by mass spectrometry.

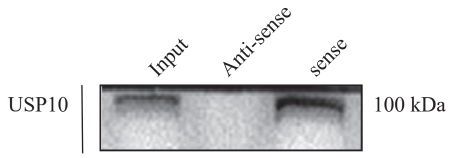

Supplementary Fig. 5 **Western blotting analysis of USP10 enriched by piR-61298 probes in LoVo cells.** The representative immunoblot image from three independent experiments is shown.

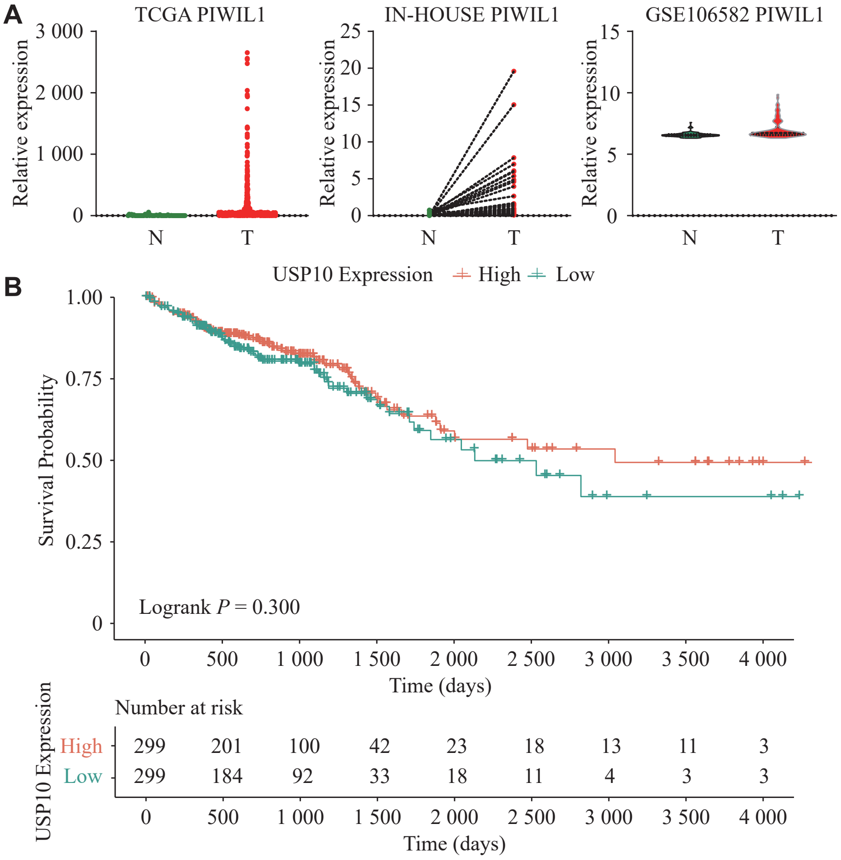

Supplementary Fig. 6 **Data analysis of piR-61298 binding protein.** A: PIWIL1 expression levels in colorectal tumor (T) tissues and normal (N) tissues from TCGA, GEO, and in-house datasets. B: Kaplan-Meier survival analysis of USP10 in colon cancer patients. Differences between two groups were analyzed using Student's *t*-test. Survival curves were analyzed using the Kaplan–Meier method with the log-rank test. Abbreviations: GEO, Gene Expression Omnibus; TCGA, The Cancer Genome Atlas; USP10, ubiquitin-specific peptidase 10.

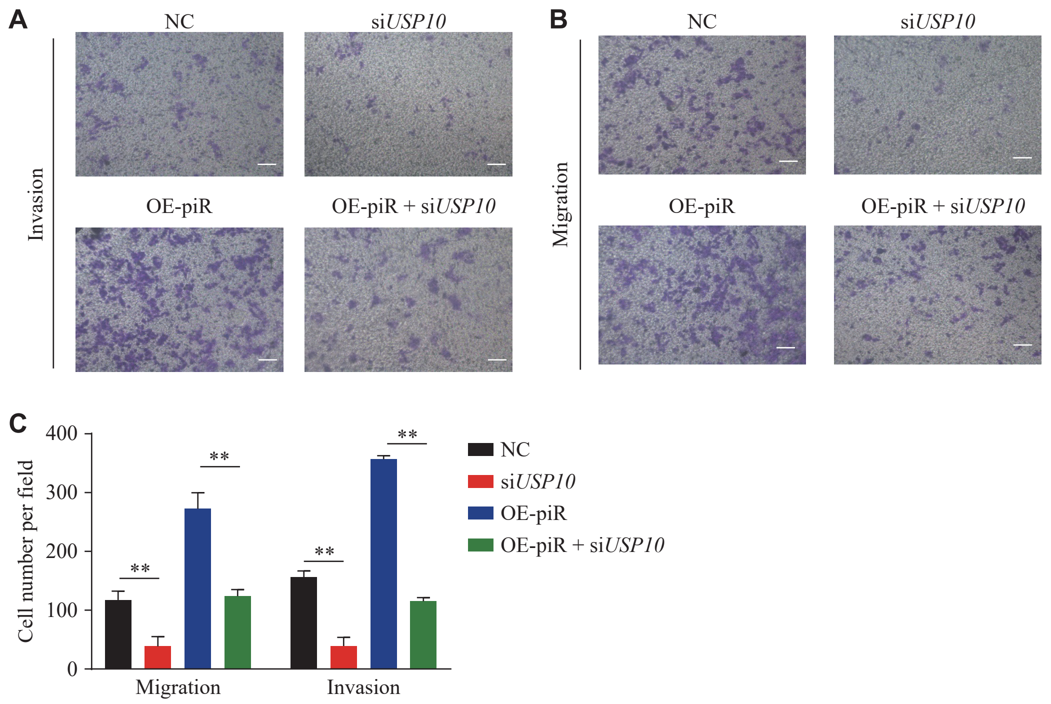

Supplementary Fig. 7 **Identification of the oncogenic role of USP10.** A and B: Effects of USP10 expression on the invasion (A) and migration (B) of LoVo cells. Scale bar, 100 µm. C: Quantification of cell number per field for invasion and migration. Statistical analyses for panel C were performed using the Kruskal–Wallis test for multiple-group comparisons, followed by pairwise Mann–Whitney *U* tests with Bonferroni correction when appropriate. Data are presented as mean ± standard deviation (n = 3); $^{**}P < 0.01$.

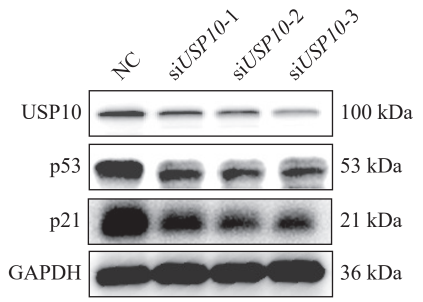

Supplementary Fig. 8 **Western blotting analysis of p53 and p21 protein levels following *USP10* knockdown.**

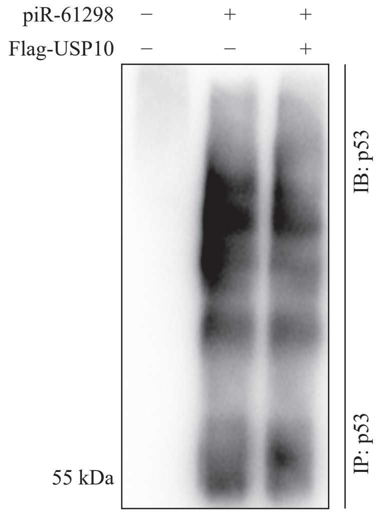

Supplementary Fig. 9 **Co-immunoprecipitation and Western blotting analyses were performed to measure the ubiquitination of p53 after the combined action of piR-61298 and USP10.**
